# Mechanistic target of rapamycin (mTOR): a potential new therapeutic target for rheumatoid arthritis

**DOI:** 10.1186/s13075-023-03181-w

**Published:** 2023-10-02

**Authors:** Fen Zhang, Ting Cheng, Sheng-Xiao Zhang

**Affiliations:** 1 Department of Rheumatology, The Second Hospital of Shanxi Medical University, No. 382, Wuyi Road, Xinghualing District, Taiyuan, 030001 Shanxi Province China; 2 Shanxi Provincial Key Laboratory of Rheumatism Immune Microecology, Taiyuan, Shanxi Province China; 3 Key Laboratory of Cellular Physiology at Shanxi Medical University, Ministry of Education, Taiyuan, Shanxi Province China

**Keywords:** Rheumatoid arthritis, mTOR, Treatment

## Abstract

Rheumatoid arthritis (RA) is an autoimmune disease characterized by systemic synovitis and bone destruction. Proinflammatory cytokines activate pathways of immune-mediated inflammation, which aggravates RA. The mechanistic target of rapamycin (mTOR) signaling pathway associated with RA connects immune and metabolic signals, which regulates immune cell proliferation and differentiation, macrophage polarization and migration, antigen presentation, and synovial cell activation. Therefore, therapy strategies targeting mTOR have become an important direction of current RA treatment research. In the current review, we summarize the biological functions of mTOR, its regulatory effects on inflammation, and the curative effects of mTOR inhibitors in RA, thus providing references for the development of RA therapeutic targets and new drugs.

## Introduction

Rheumatoid arthritis (RA) is a chronic autoimmune disease characterized by erosive arthritis and a pathological basis of synovitis, which causes morning stiffness, joint pain, and impairment of movement functions [1]. Extraarticular symptoms of severe active RA generally occur in the cardiovascular, respiratory, blood, and urinary system and may cause other comorbidities [2]. The global incidence of RA is approximately 1%, and two thirds of the cases are women [3]. At the joint inflammatory site, abnormal modulation of mTOR signaling results in continuous feedback between stromal cells and infiltrating immune cells, leading to persistent inflammation and tissue damage, driving the pathological tissue refactoring and eventually causing organ dysfunction [4]. Recent studies reported that activation of the mechanistic target of rapamycin (mTOR) affects the abundance and functioning of immune and stromal cells and may thus be an essential pathway in the pathogenesis of RA. In this review, we describe the structure and processes of the mTOR pathway and highlight its role in the pathogenesis of RA to provide a reference for the development of novel clinical treatment avenues.

### The biology of mTOR

In 1975, Sehgal et al. [5] discovered an antifungal macrolide antibiotic produced by the bacterium *Streptomyces hygroscopicus*, which was termed rapamycin. Rapamycin binds to the 12-kD intracellular protein FKBP12, thus forming a high-affinity complex with the protein FK506 [6]. The FKBP12-rapamycin complex then fuses with the mTOR protein, which was first isolated from mammalian tissue [7]. mTOR is a highly conserved serine/threonine protein kinase that regulates cell proliferation, differentiation, and angiogenesis [8].

### mTOR complexes

**Fig. 1:**
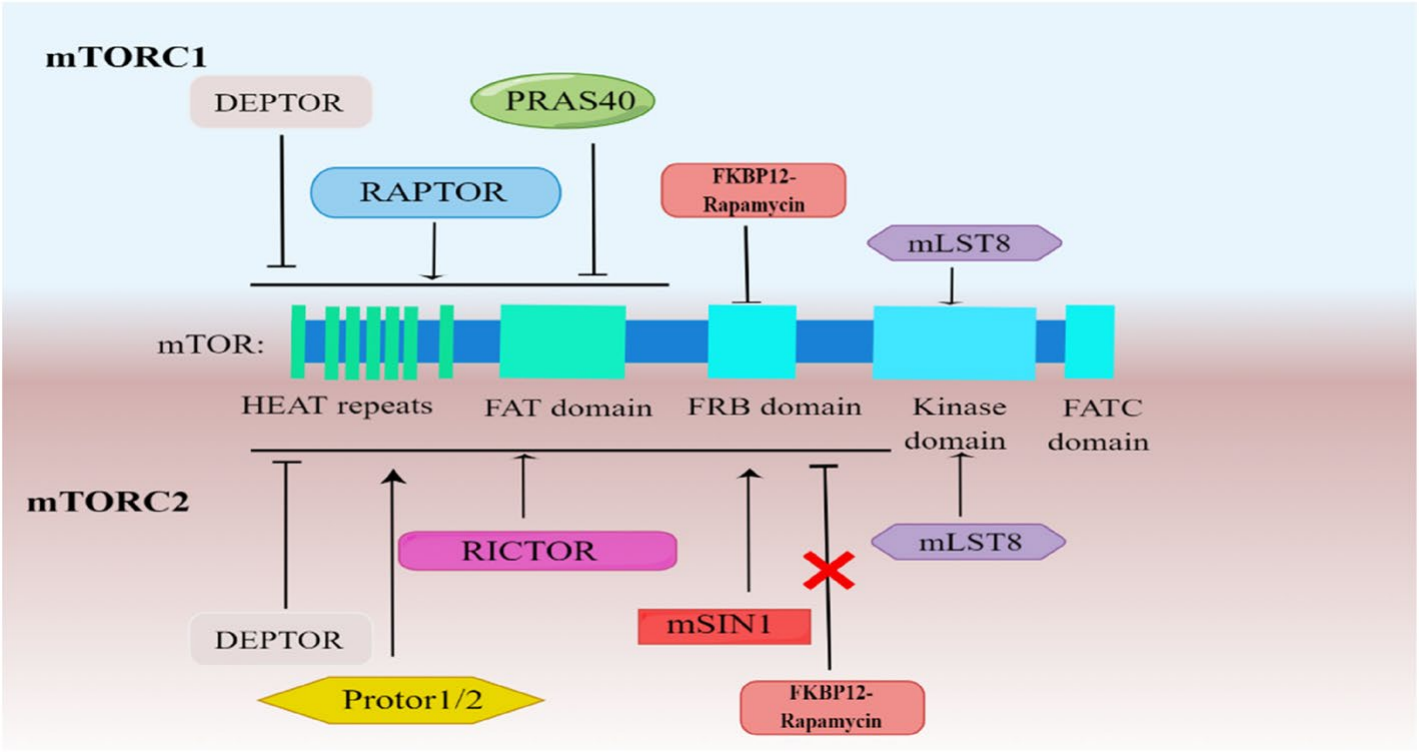
The mTORC1 and mTORC2 complex. The FKBP12-rapamycin complex binds to the FK506-binding protein 12 and inhibits mTORC1 by blocking its association with the FRB domain. This leads to steric hindrance and prevents the activation of mTORC1. On the other hand, mTORC2 is resistant to rapamycin due to the presence of rictor and msin1, which block the binding of the FKBP12-rapamycin complex to the FRB domain of mTORC2. In the mTORC1 complex, raptor plays a crucial role in promoting the phosphorylation of various downstream targets. mlst8 stabilizes the mTORC1 complex, whereas deptor negatively regulates its activity. pras40 also interacts with mTORC1. In the mTORC2 complex, rictor is an essential component that helps stabilize the complex while msin1 contributes to its regulation. This figure provides insight into the structural components and interactions within mTORC1 and mTORC2 complexes, highlighting their sensitivity or insensitivity to the FKBP12-rapamycin complex and the respective roles of key proteins. raptor, regulatory-associated protein of TOR; mlst8, mammalian lethal with sec-13 protein 8; deptor, DEP domain–containing mTOR interacting protein; pras40, proline-rich Akt substrate of 40kD; rictor, rapamycin-insensitive companion of mTOR; msin1, stress-activated protein kinase interacting protein 1

mTOR forms two functionally distinct complexes with proteins: mTOR complex (mTORC)1 and mTORC2 [9]. mTORC1 comprises mTOR, regulatory-associated protein of TOR (raptor), mammalian lethal with sec-13 protein 8 (mlst8), DEP domain–containing mTOR interacting protein (deptor), and proline-rich Akt substrate of 40kD (pras40) [8, 10]. mTORC2 is composed of mTOR, rapamycin-insensitive companion of mTOR (rictor), stress-activated protein kinase interacting protein 1 (msin1), mlst8, deptor, and protor-1/2 [11–13]. mTOR bears several conserved domains, including the N terminus HEAT repeats, FAT domain, FRB domain, Kinase domain, and the C-terminal FATC domains [14] (Fig. 1).

In more detail, the FKBP12-rapamycin complex binds to the FRB domain of mTORC1 to produce steric hindrance, which interferes with interactions between the substrate and the active site of mTORC1 [15, 16]. Furthermore, mTORC1 promotes cell growth by controlling glycolysis, upregulating mRNA transcription, and synthesizing and breaking down proteins and lipids [17]. In addition, mTORC1 regulates catabolic processes including autophagy [18]. Unlike mTORC1, in mTORC2, rictor and msin1 block the binding of the FKBP12-rapamycin complex to the FRB domain of mTORC2, thus revealing the potential mechanism underlying the insensitivity of mTORC2 to rapamycin (Fig. 1) [19, 20]. mTORC2 regulates cell survival, migration, metabolism, and cytoskeleton arrangement through molecular networks [21].

### Upstream regulators of mTOR

Several ligands activate mTOR signaling, including growth factors, amino acids, antigens, and cytokines [22]. The pathways of mTORC1 activation have been extensively researched; however, mTORC2 regulation is currently less understood. Compared to mTORC1, mTORC2 is an insensitive signaling factor [8].

**Fig. 2:**
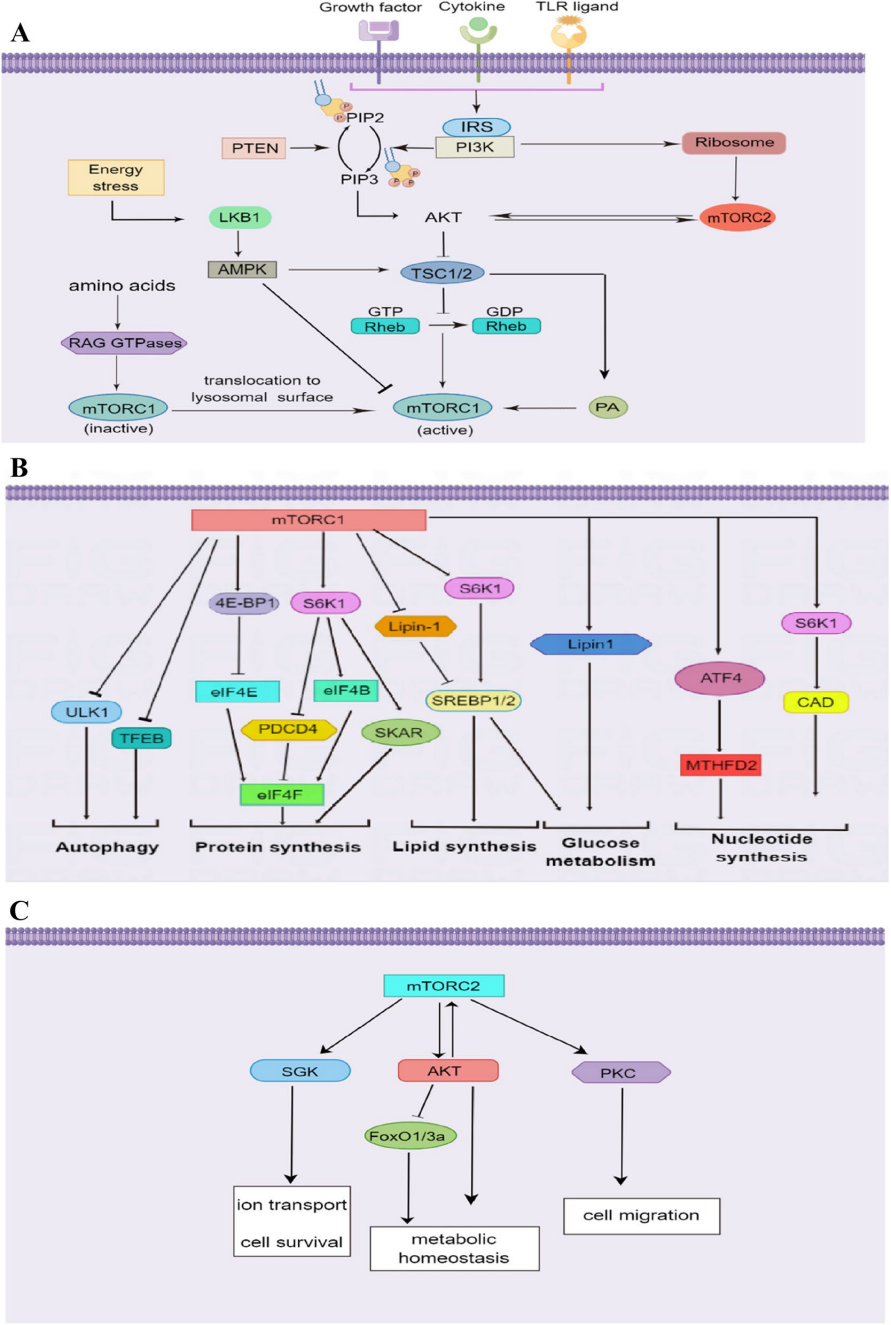
**A** The upstream pathway of mTORC1 and mTORC2. Growth factor, cytokines, or TLR signaling activates PI3K, which in turn activates AKT. The AMPK, LKB1, and IRS are also involved in regulating mTORC1 and mTORC2. TLR, Toll-like receptor; PI3K, phosphatidylinositol-3-OH kinase; AKT, RAC-α serine/threonine-protein kinase; TSC, tuberous sclerosis complex; Rheb, Ras homolog enriched in the brain; PTEN, phosphatase and tensin homolog deleted on chromosome 10; PIP2, ephosphatidylinositol-3,4-bisphosphate; PIP3, phosphatidylinositol-3,4,5-triphosphate; AMPK, the mitogen-activated protein kinase; LKB1, liver kinase B1, IRS, insulin receptor substrate. **B** The downstream pathway of mTORC1. mTORC1 activates S6K1 and eIF4E to promote protein synthesis and cell growth. mTORC1 also inhibits 4E-BP1 to initiate translation. Additionally, mTORC1 influences PDCD4, SKAR, ATF4, MTHFD2, CAD, HIF1α, ULK1, and TFEB to regulate glucose metabolism, lipid synthesis, and autophagy. S6K1, S6 kinase 1; eIF4E, eukaryotic translation initiation factor 4E; eIF4B, eukaryotic translation initiation factor 4B; 4E-BP1, the eIF4E binding protein 1; PDCD4, programmed cell death protein 4; SKAR, S6K1 Aly/REF-like target; ATF4, activating transcription factor 4; MTHFD2, methylenetetrahydrofolate dehydrogenase 2; CAD, carbamoyl-phosphate synthetase; HIF1α, hypoxia-inducible factor-1α; ULK1, unc-51-like kinase1; TFEB, transcription factor EB. **C** The downstream pathway of mTORC2. mTORC2 activates SGK1 and PKCα, which play roles in cell survival and cytoskeletal organization. SGK1, serum and glucocorticoid inducible kinase 1; PKCα, protein kinase C-α

The activity of mTORC1 is primarily mediated by phosphatidylinositol-3-OH kinase/RAC-α serine/threonine-protein kinase/tuberous sclerosis complex (PI3K/AKT/TSC) and liver kinase B1/the mitogen-activated protein kinase (LKB1/AMPK) axis. Growth factor receptor, cytokines, and Toll-like receptor (TLR) ligands regulate mTORC1 activity by the PI3K/AKT/TSC signaling pathway. They phosphorylate PI3K to activate downstream effector AKT, then restrain the TSC1/TSC2 complex [23]. Ras homolog enriched in the brain (Rheb) primarily occurs in a GTP-bound activated state [14, 24]. Inactivation of the TSC complex restrains the hydrolysis of Rheb-GTP, preventing it from conversion into Rheb-GDP. Finally, Rheb-GTP directly activates mTORC1 [25]. Through continuously improving understanding of the pathway, phosphatase and tensin homolog deleted on chromosome 10 (PTEN) was found to neutralize the activity of PI3K by dephosphorylating the downstream products of PI3K, which include ephosphatidylinositol-3,4-bisphosphate (PIP2) and phosphatidylinositol -3,4,5-triphosphate (PIP3) [14, 26]. The most remarkable characteristic of mTORC1 is its activating ability on the surface of the lysosomal membrane [27]. Growth factors activate Rheb, and amino acids recruit mTORC1, both of which are indispensable for the activation of mTORC1. AMPK regulates glucose metabolism and perceives changes in ATP levels [28, 29]. Energy stress activates LKB1-dependent AMPK, which phosphorylates downstream TSC2, then promotes the formation of the TSC1/TSC2 complex, and further inhibits the activity of mTORC1 [30]. In addition, AMPK directly phosphorylates raptor and suppresses mTORC1 signaling [31] (Fig. 2A).

The activity of mTORC2 is mainly regulated through the PI3K/AKT axis. In detail, PI3K induces PtdIns(3,4,5)P(3) to combine with the pleckstrin homology domain of msin1, blocking inhibition of the mTOR kinase domain by msin1, thereby activating mTORC2 [32, 33]. PI3K also promotes the combination of the ribosome and mTORC2, and the ribosome is necessary for inducing mTORC2 kinase activity [34]. Of note, partial activation of Akt boosts the activation of mTORC2, which phosphorylates and ultimately activates Akt, resulting in a positive feedback loop [35] (Fig. 2A).

### Downstream targets of mTOR

mTORC1 maintains metabolic homeostasis by regulating biological synthesis and catabolic processes. Activated mTORC1 phosphorylates its downstream targets S6 kinase 1 (S6K1) and the eIF4E binding protein 1 (4E-BP1) to regulate protein synthesis [36]. Phosphorylated 4E-BP1 releases cap-binding protein eukaryotic translation initiation factor 4E (eIF4E), which counteracts inhibition of protein synthesis by enabling eIF4E to form the eIF4F complex and to participate in cap-dependent translation [37, 38]. S6K1 regulates eukaryotic translation initiation factor 4B (eIF4B), programmed cell death protein 4 (PDCD4) [39], and S6K1 Aly/REF-like target (SKAR) [40] to participate in protein synthesis. eIF4B is a positive regulator of eIF4F complex, and PDCD4 is a negative regulator of eIF4A [41, 42]. The mTORC1 phosphorylates lipin 1 to increase the activity of SREBP1 or activates SREBP1 through an S6K1-dependent mechanism, thus participating in lipid synthesis [43, 44]. In addition, mTORC1 promotes nucleotide synthesis by stimulating the mTHF cycle, which increases ATF4 levels to upregulate the expression of methylenetetrahydrofolate dehydrogenase 2 (MTHFD2) [45]. Moreover, S6K1 phosphorylates carbamoyl-phosphate synthetase (CAD) to activate the de novo pyrimidine synthesis pathway [46]. Furthermore, mTORC1 increases the expression of transcription factor HIF1α to promote glucose metabolism [44]. SREBP1 is also involved in the regulation of the pentose phosphate pathway. Taken together, mTORC1 regulates various metabolic pathways to coordinate anabolism (Fig. 2B).

mTORC1 negatively regulates catabolic processes such as autophagy to promote cell growth [17, 47]. Under nutrient sufficiency, activated mTORC1 phosphorylates and suppresses unc-51-like kinase1 (ULK1) [48]. Transcription factor EB (TFEB) regulates the expression of autophagy and lysosomal genes, which is also phosphorylated and inhibited by mTORC1 to regulate autophagy indirectly [49] (Fig. 2B).

Primary downstream targets of mTORC2 include AKT [50], serum and glucocorticoid inducible kinase 1 (SGK1) [51], and protein kinase C-α (PKCα) [11] (Fig. 2C). PKC-α regulates the actin cytoskeleton to adjust cell shape [52]. Activated SGK1 controls ion transport and cell survival [51]. Additionally, Akt phosphorylates and inhibits transcription factor FoxO1/3a to promote metabolic homeostasis [53].

### The role of mTOR in the pathogenesis of RA

**Fig. 3:**
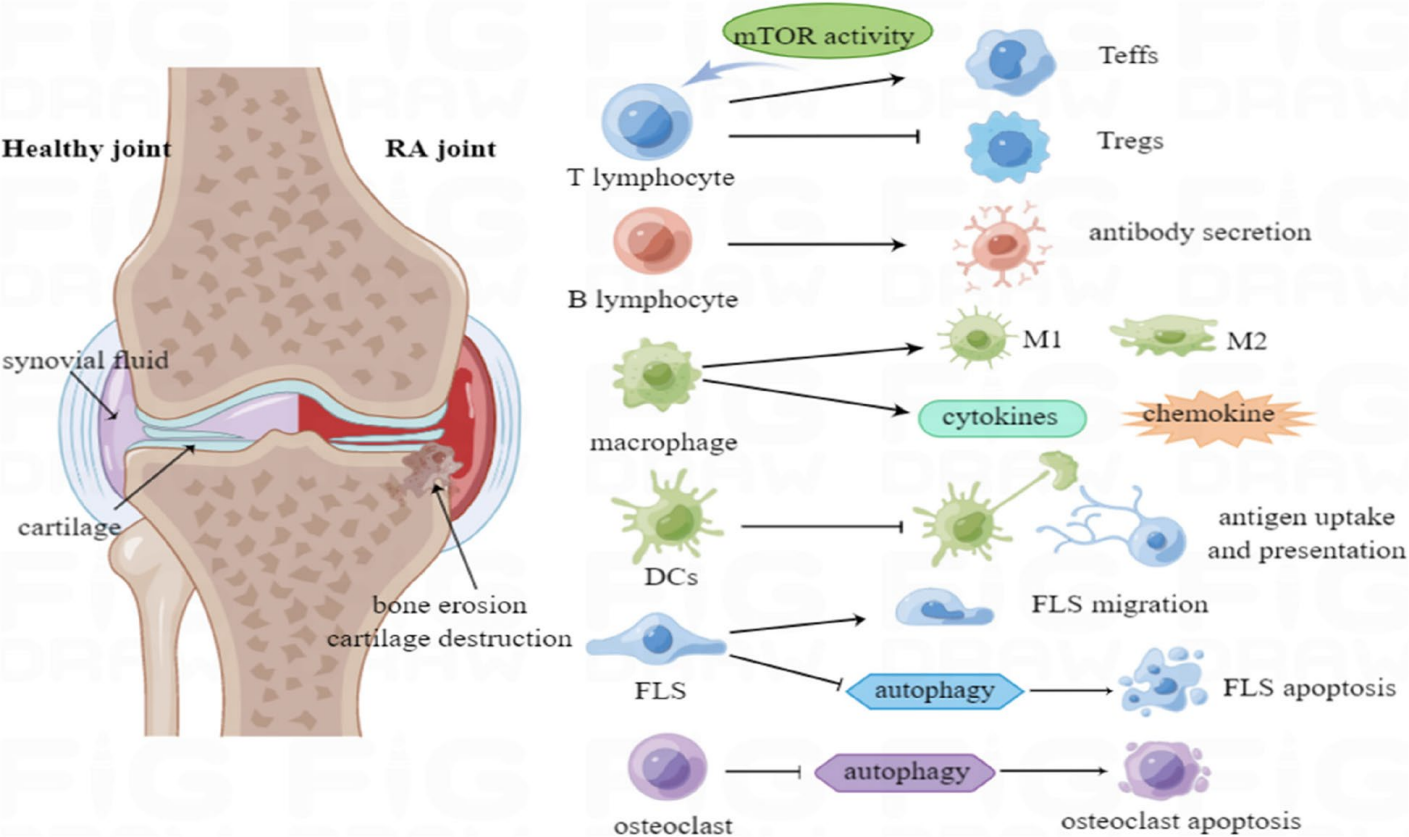
Inflammation in RA-affected joints. The pathogenesis of RA reflects the complex interactions between various cell groups in the synovium, mediated by direct contact between cells and various types of secreted or exfoliated molecules. mTOR signaling controls the recruitment and activation of innate, acquired immune cells and FLSs during RA, thus producing numerous chemokines, pro-inflammatory cytokines, and cathepsin to degrade the extracellular matrix and cartilage, further contributing to the early characteristics of synovitis

The pathogenesis of RA reflects the complex interactions between various cell groups in the synovium, mediated by direct contact between cells and various types of secreted or exfoliated molecules. mTOR signaling controls the recruitment and activation of innate, acquired immune cells and fibroblast-like synoviocytes (FLSs) during RA, resulting in the production of numerous chemokines, pro-inflammatory cytokines, and cathepsin to degrade extracellular matrix and cartilage, further contributing to the early characteristics of synovitis [3, 54] (Fig. 3).

### mTOR regulates proliferation and differentiation of T lymphocytes

T lymphocytes play crucial roles in the progression of RA. Effector T cells (Teffs) such as Th1, Th2, and Th17 cells stimulate monocytes or macrophages to synthesize pro-inflammatory cytokines, leading to destructive changes in RA-affected joints [55]. By contrast, regulatory T cells (Tregs) promote the secretion of inhibitory cytokines to inhibit the proliferation and activation of autoreactive T cells and maintain immune homeostasis [56]. Thus, a disturbed Teffs/Tregs balance determines the progression of RA [57]. mTOR signaling is essential for the proliferation and activation of T cell lineages, and mTORC1 and mTORC2 make distinct respective contributions [58, 59].

mTORC1 regulates HIF1α to promote the change from oxidative phosphorylation to glycolysis, thereby supporting the differentiation of T lymphocytes [60]. Increased glycolysis promotes T cells to differentiate into Th1, Th2, and Th17 cells [61]. By contrast, deprivation of glycolysis promotes the development of Tregs [61]. Glycolysis activates mTORC1 to block the expression of FOXP3 and affects the function of Tregs [62]. Leucine or serine deficiency can inhibit the activity of mTORC1 and restrict the differentiation of T cells into Teffs [63]. Indoleamine 2,3-dioxygenase negatively regulates mTORC1 signal transduction via PTEN, preventing proliferation of Teffs and promoting induction of Tregs [62, 64]. De novo fatty acid synthesis inhibits differentiation of Th17 cells and stimulates expansion of Tregs [65]. By contrast, mTORC2 regulates the activity of RhoA to accelerate Th2 cell differentiation [66]. Consequently, mTORC1-deficient T cells cannot differentiate into Th1 or Th17 cells, and mTORC2-deficient T cells lack the ability of Th2 differentiation [66, 67]. Further mTORC1 and mTORC2 negatively regulate the differentiation and functioning of Tregs [68].

### mTOR regulates the proliferation and activation of B lymphocytes

B cells participate in the pathogenesis of RA by producing autoantibodies, proinflammatory cytokines, and chemokines, acting as efficient antigen-presenting cells and regulating T cell activation and differentiation [69, 70]. mTOR signaling pathway regulates immune function and homeostasis of peripheral B cell in RA [71].

Inhibition of the mTOR pathway in B cells restrains the growth of mitogen-dependent embryonic cells and suppresses cell proliferation by blocking cell cycle progression in the G1 phase, which markedly inhibits the differentiation and secretory functions of B cells. mTOR acts as an essential factor in B cell lineage differentiation; for example, mTOR negatively regulates the development of memory B cells [72]. Activated mTORC1 combines with Syk and induces EZH2 expression to inhibit BACH2 transcription, resulting in increased expression of Blimp-1 and XBP1, which are essential for differentiation and antibody secretion of plasma B cells [73–75]. mTORC1 signaling is critical for the early development of B cells and primary or secondary immune responses to T-dependent antigens. However, excessive activation of mTORC1 is pernicious to B cell maturation, especially with respect to marginal zone B cells [76, 77].

mTORC2 signaling regulates PI3K signaling in early B cells to upregulate IL-7R signaling and Rag gene expression, and pathway disruption results in interrupted B cell development [76]. Moreover, mTORC2 regulates the expression of NF-κB target genes, which affects cell survival downstream, mediated by BCR and BAFF-R signaling, and also influences antibody production in rictor-deficient mice [78]. BAFF-R plays a role in the activation of PI3K signaling pathways, as well as classical and alternative NF-κB signaling networks. The alternative NF-κB signal pathway involves phosphorylation of IKK1 by NF-κB -induced kinase (NIK), and IKK1 phosphorylates mTOR, leading to the transduction of downstream signaling. Moreover, BAFF-R is also involved in the induction of PI3K signaling, which enhances the activity of mTORC1 [79]. In addition, IL-27 has been identified as the critical driver of mTOR activation in B cells during RA. The mTOR inhibitor effectively restores IL-27-induced excessive activation, proliferation, and secretion of B cells and the abnormal ratio of regulatory B cells to plasma cells in vivo and in vitro [71]. Therefore, the mTOR pathway plays a crucial role in B cell immune dysfunctions during RA.

### mTOR regulates macrophage activation, polarization, and migration

Macrophages are the first line of defense against pathogen invasion and the principal response to infection [80]. The normal synovial membrane is divided into the lining and synovial sub-lining layers. The lining layer includes macrophage-like synoviocytes (MLS) and FLSs. The sub-lining comprises synovial macrophages (SMs), a network of vascular capillaries, and fibrous and adipose tissue [81]. The occurrence of abundant activated macrophages in the synovia is an early marker of RA. M1 secretes pro-inflammatory cytokines (mainly IL-1, IL-6, and TNF), which lead to osteoclast (OC) activation and joint erosion, and M2 produces anti-inflammatory cytokines such as IL-10 and transforming growth factor-β (TGF-β) to promote angiogenesis and tissue remodeling [82, 83]. M1 macrophages show high expression of MHCII and costimulatory molecules such as CD80 and CD88, which are the basis of effective T cell antigen presentation. M1 macrophages in RA synovium also secrete multiple chemokines to promote the recruitment of macrophages or neutrophils in arthritic sites, which corresponds to clinical severity of RA [84]. Taken together, during RA, macrophages produce proinflammatory cytokines, chemokines, and free radicals to activate immune and non-immune cells, leading to joint inflammation and destruction.

The essential roles of the mTOR pathway in macrophage biology have been partially elucidated over the past few years. Macrophage function depends on activation and polarization into subtypes with distinct effector functions. Macrophage polarization is regulated by various factors, including cytokines, growth factors, and environmental stimuli. Therefore, the mTOR pathway changes environmental and metabolic factors to influence the polarization of macrophages [85]. Inhibition of mTORC1 enhances the polarization of M1 macrophages, whereas TSC1 deletion increases the activity of mTORC1 and reduces mTORC2 activity to promote the polarization of M1 and decrease M2 macrophage polarization [86, 87]. In addition, differences of AKT subtypes control macrophage polarization [88]. AKT1 deletion promotes M1 macrophages, and AKT2 ablation produces M2 macrophages. Consistent with this, AMPK deletion decreases the activation of AKT and promotes M1 macrophage polarization [89]. With regard to metabolism functions, the activation of pyruvate dehydrogenase kinase 1 (PDK1) promotes M1 macrophage polarization, and aerobic glycolysis stimulator MYC polarizes M2 macrophages [90]. Overall, macrophage polarization is controlled by the mTOR pathway; however, the role of individual pathway members requires further research. mTORC1 inhibits the migration of macrophages and reduces the migratory activity of immune cells, and activation of mTORC1 induces the translation conversion of transcription factors to reduce the expression of chemokines CCL2, CCL3, and CCL4 [91]. In addition, it has been confirmed that the arthritogenicity of RA induced by Syntenin-1 relies on the remodeling of macrophages activated by mTOR and their capacity to cross-regulate Th1 cells through induction of IL-12 and IL-18. This interaction closely associated with mTOR's regulation of T cell and macrophage differentiation, and holds significant implications for guiding future research in this field [92].

### mTOR regulates dendritic cell (DC) differentiation, maturation, and function

DCs are efficient antigen-presenting cells that initiate the initial immune response. During infection or under stressful conditions, mature DCs present antigens to naive T cells, resulting in the differentiation of effector CD4^+^T cells, production of B cell antibodies, and activation of macrophages [93]. DCs also contribute significantly to maintaining immune homeostasis and tolerance [94]. DCs secrete chemokines to attract macrophages, neutrophils, and T cells to the synovium during RA, which potentiates subsequent immune responses [95]. The specific mechanisms of various pro-inflammatory immune cells are described in detail above. DCs are directly or indirectly involved in the development of RA, and interactions between DCs and Tregs also interfere with RA development. Tregs express cytotoxic T-lymphocyte-associated protein-4, lymphocyte-activating gene-3, and neuropilin-1 to suppress DC functions [96].

The suppressive effects of mTOR inhibition on DCs have thoroughly investigated. Inhibition of mTOR reduces the expression of antigen uptake receptors and costimulatory molecules in DCs and restrains receptor-mediated phagocytosis [97]. Furthermore, inactivation of mTOR was suggested to inhibit CD86 expression induced by TLR ligands (such as LPS) or CD40-specific during DCs differentiation [98]. mTOR inhibitors attenuate the HIF-1α pathway to suppress hypoxia-induced inflammation and affect the differentiation of immature DCs [99]. mTOR is induced by Flt3 ligand (Flt3L) and is necessary for the Flt3L-driven development of DCs [100], and mTOR inhibits IL-1β production to impede DC maturation and the ability to stimulate effector T cell responses. In contrast, DCs under mTOR inactivation conditions favor differentiation of FOXP3^+^ Tregs [98]. In conclusion, mTOR exerts multiple effects on DC differentiation and functioning, which interferes with antigen uptake and presentation, and it affects cytokine production and chemokine receptor expression to modulate immune responses.

### mTOR regulates the proliferation of RA FLSs

Abnormal proliferation, as well as high invasion and migration of FLSs in joints, are the main characteristics of RA [101]. Inflammation of RA-affected joints commences with FLS proliferation and invasion of immune cells into the synovium. FLSs then elicit overproduction of chemokines, cytokines, and matrix degradation molecules, thereby promoting immune cell infiltration and cartilage degradation [102]. Additionally, activated FLSs migrate to the synovial sub-lining layer to promote angiogenesis and exacerbate synovial hyperplasia and bone destruction [103]. In RA synovial cells, the mTOR signaling pathway is abnormally activated to participate in the regulation of RA FLS invasion [104]. Aberrant activation of PI3K/Akt/mTOR leads to high expression of anti-apoptosis genes, reduced autophagy, and continuous proliferation of FLSs [105, 106]. Inactivating mTOR prevents FLSs from recombining the actin cytoskeleton and eliciting bone destruction [104].

### mTOR regulates differentiation and formation of OCs

OCs, differentiated giant multinucleated cells derived from the monocyte/macrophage lineage, decompose the bone matrix by producing various enzymes and acids [107]. OCs are hyperfunctioning during RA, resulting in bone destruction. mTOR controls the autophagy pathway to participate in OC differentiation and formation [108]. Inhibition of autophagy blocks the differentiation of RA mouse macrophages into OCs, decreases bone erosion, and reduces OC abundances. Conditional deletion of mTOR (raptor) in OCs results in decreased OC differentiation and activity. The increased expression of structurally active S6K1 rescues damage of OCs differentiation under Raptor-deficiency [109]. The AMPK/mTOR/p70S6K signaling pathway induces autophagy to inhibit OC differentiation, thus regulating bone mineral density and improving bone mass [110]. Furthermore, inhibition of the AMPK/mTOR/ULK1 pathway reduces autophagy in OCs exposed to high levels of glucose [111], and activation of the PI3K/AKT/mTOR axis suppresses autophagy of OCs treated with hydrogen sulfide [108]. Additionally, pharmacological inhibition of mTOR induces OC apoptosis and inhibits OC activity and differentiation [112, 113]. In conclusion, elucidating the mTOR pathway provides essential insights into the molecular mechanism of regulating OCs.

### mTOR-targeted therapy

As described above, mTOR regulates many cellular processes and participates in the development of RA. Blocking this pathway through mTOR inhibitors is beneficial for RA treatment through changes in the immune and metabolic environment.

### Rapamycin

Rapamycin is a potent mTOR inhibitor that controls antigen-induced T-cell expansion, antibody production, and cellular proliferation [114]. This compound was originally approved by regulatory authorities in 1999, with the purpose to prevent kidney allograft rejection. Further research revealed that mTOR is generally activated in neoplasms and controls cancer cell metabolism by regulating key metabolic enzymes; thus, it has been targeted for cancer treatment [115]. Moreover, rapamycin inhibits the proliferation of endothelial cells and prevents the deterioration of disease by intra-arterial drug-eluting stents [116]. In short, rapamycin has been successfully used for numerous medical applications, such as anti-inflammation, anti-immune rejection, anti-tumor, and anti-endothelial proliferation treatments, and it plays a key role in the treatment of many diseases.

Rapamycin significantly inhibits the activity of arthritis and improves immune function in RA mice [117, 118]. Rapamycin was also successfully used in clinical trials for RA treatment [119–121]. For instance, Wen et al. [120] found that patients receiving rapamycin therapy achieved profound clinical improvement through increased circulating Tregs during RA. In addition, rapamycin reduces the secretion of inflammatory cytokines such as IL-6, TNF, and IL-1β, relieving the symptoms of RA [122]. Moreover, rapamycin also decreases the necessity for conventional disease-modifying antirheumatic drugs in controlling RA activities [120]. Taken together, rapamycin induces autoimmune tolerance to reduce joint inflammation and is expected to be a new option for treating RA.

### Rapalogues

Semi-synthetic rapamycin analogs are collectively referred to as rapalogues, such as everolimus (also known as RAD001), temsirolimus (CCI779), and ridaforolimus [123–125]. Compared with rapamycin, rapalogues are designed for higher water solubility and oral administration [126]. Everolimus inhibits the proliferation of synovial cells and the activity of OCs, which affects bone erosion during RA [112, 127]. Everolimus has the advantages of a simple administration route (oral), low cost, and low risk of infection [128]. A multicenter, randomized, double-blind trial investigating the safety and efficacy of everolimus reported that everolimus plus methotrexate showed better clinical efficacy and reduced adverse reactions [129]. Temsirolimus neutralizes the stimulation of LAT1 by IL-17 and reduces leucine uptake and fibroblast migration to prevent further erosion of the cartilage and bone [130].

### Second-generation mTOR inhibitors

Dual PI3K and mTOR inhibitor NVP-BEZ23527 decreases mTOR and Akt phosphorylation to accelerate the apoptosis of bone cells. Furthermore, the dual effects of BEZ235 on PI3K/Akt and mTOR signaling pathways inhibit the activation of fibroblasts and eliminate the defect of rapamycin in p-Akt feedback activation of Ser473 after treatment with TGF-β [131]. Clinical trials with other dual mTOR/PI3K inhibitors, such as GSK2126458, PI103, SF1126, and GSK2126458, for the treatment of rheumatic diseases are underway [132].

### N-acetylcysteine (NAC)

NAC is an antioxidant and anti-inflammatory agent whose functions are to promote glutathione biosynthesis and scavenge free radicals [133]. Glutathione modulates T-cell differentiation by regulating mitochondrial transmembrane potential (Δψm) and mTOR [134]. During RA, oxidative stress stimulates OC formation and activation to promote bone resorption. NAC reduces IL-17-induced activation of the mTOR/JNK/NF-κB (nuclear factor κB) pathway to regulate the expression of RANKL in synovial fibroblasts and osteoblasts, preventing inflammation and bone destruction during RA [135]. NAC supplementation improves clinical indicators of RA [136]. Additionally, continued exposure of T cells at sites of inflammation elicits high levels of reactive oxygen species (ROS), which results decreased intracellular levels of GSH, dysregulation of redox balance, impaired signal transduction of the TCR/CD3 complex, and ultimately to synovial T cell hyporesponsiveness during RA [137]. Accumulation of ROS induces oxidative damage and chondrocyte senescence, thereby accelerating the degeneration of the cartilage matrix, and antioxidant NAC reverses this process [138]. NAC effectively scavenges free-oxygen radicals, decelerates the process of cartilage degradation, reduces synovitis, and relieves pain [139]. Furthermore, NAC prevented chondrocyte apoptosis and cartilage destruction in an experimentally induced rat model of RA [140]. However, long-term oral NAC administration is associated with higher risk of RA [141], and potential clinical application of NAC requires further study.

### Metformin (Met)

Met is the cornerstone of diabetes treatment, and during treatment of rheumatic diseases, it activates AMPK and inhibits mTORC1 [142–144]. Met-mediated AMPK activation and inhibition of mTOR activity regulate autophagy flux, inhibit NF-κB signal transduction and production of inflammatory cytokines, and reduce inflammation during experimentally induced arthritis [145, 146]. Met effectively inhibits the proliferation of RA-FLS by inducing G2/M cell cycle phase arrest, thus upregulating and downregulating phosphorylation of p70S6K and 4E-BP1 [147]. Furthermore, Met exerts an immunomodulatory effect of collagen induced arthritis by inhibiting Th17 cell differentiation and upregulating Treg differentiation [148]. Met inhibits differentiation of B cells into plasma cells and formation of spontaneous germinal center through the AMPK/mTOR/STAT3 signal pathway and thus reduces autoantibodies production and inflammation [149]. A retrospective cohort study found that long-term use of Met is related to reduced risk of developing RA [150].

### Statins

Statins are lipid-lowering drugs that prevent cholesterol synthesis by inhibiting 3-hydroxy-3-methylglutaryl-CoA reductase, commonly used in treating hypercholesterolemia and cardiovascular-related diseases [151]. Statins are also used as immunomodulators to block the adhesion of antigen-presenting cells to T cells, thus preventing the proliferation and function of T cells. Statins influence the activation of GTPases, such as Rho-GTPases, thereby regulating the transduction of PI3K/Akt/mTOR and ERK signaling pathways. The effects of statins on Tregs in RA patients in vivo and in vitro confirm that Tregs participate in the immunomodulatory impact of statins on RA [152].

## Conclusion

During the pathogenesis of RA, the mTOR pathway is activated by multiple antigens, resulting in the recruitment and differentiation of immune cells and the activation of synovial and osteoclastic cells. Current studies suggest that targeting the mTOR pathway holds promise as a treatment for RA. However, indiscriminate suppression of mTOR to prevent RA may have unintended consequences. Long-term immunosuppressive therapy can compromise immune defense and surveillance, thereby increasing the risk of infections and tumors. Therefore, it is crucial to clarify the precise role of mTOR pathway in different stages of RA and its interaction with other signaling pathways and develop targeted therapies that do not disrupt essential physiological processes. Future investigations should prioritize understanding the potential side effects of immunotherapy in order to ensure the safety and efficacy of mTOR-based therapy for RA.

## Data Availability

Not applicable.

## Abbreviations

RA: Rheumatoid arthritis
mTOR: Mechanistic target of rapamycin
raptor: Regulatory-associated protein of TOR
mlst8: Mammalian lethal with sec-13 protein 8
deptor: DEP domain–containing mTOR interacting protein
pras40: Proline-rich Akt substrate of 40kD
rictor: Rapamycin-insensitive companion of mTOR
msin1: Stress-activated protein kinase interacting protein 1
PI3K: Phosphatidylinositol-3-OH kinase
AKT: RAC-α serine/threonine-protein kinase
TSC: Threonine-protein kinase/tuberous sclerosis complex
LKB1: Liver kinase B1
AMPK: Mitogen-activated protein kinase
TLR: Toll-like receptor
Rheb: Ras homolog enriched in the brain
PTEN: Phosphatase and tensin homolog deleted on chromosome 10
PIP2: Ephosphatidylinositol-3,4-bisphosphate
PIP3: Phosphatidylinositol-3,4,5-triphosphate
S6K1: S6 kinase 1
eIF4E: Eukaryotic translation initiation factor 4E
eIF4B: Eukaryotic translation initiation factor 4B
4E-BP1: EIF4E binding protein 1
PDCD4: Programmed cell death protein 4
SKAR: S6K1 Aly/REF-like target
ATF4: Activating transcription factor 4
MTHFD2: Methylenetetrahydrofolate dehydrogenase 2
CAD: Carbamoyl-phosphate synthetase
HIF1α: Hypoxia-inducible factor-1α
ULK1: Unc-51-like kinase1
TFEB: Transcription factor EB
SGK1: Serum and glucocorticoid inducible kinase 1
PKCα: Protein kinase C-α
FLSs: Fibroblast-like synoviocytes
Teffs: Effector T cells
Tregs: Regulatory T cells
MLS: Macrophage-like synoviocytes
SMs: Synovial macrophages
OC: Osteoclast
TGF-β: Factor-β
DC: Dendritic cell
Flt3L: Flt3 ligand
NAC: N-acetylcysteine
NF-κB: Nuclear factor κB
ROS: Reactive oxygen species
Met: Metformin
